# Trajectory tracking for agricultural tractor based on improved fuzzy sliding mode control

**DOI:** 10.1371/journal.pone.0283961

**Published:** 2023-04-06

**Authors:** Chengqiang Yin, Shourui Wang, Jie Gao, Xiaowei Li

**Affiliations:** 1 School of Machinery and Automation, Weifang University, Weifang, Shandong Province, China; 2 School of Mechanical and Electrical Engineering, Lanzhou University of Technology, Lanzhou, Gansu Province, China; 3 Institute of Microelectronics of the Chinese Academy of Sciences, Beijing, China; 4 University of Chinese Academy of Sciences, Beijing, China; Beijing Institute of Technology, CHINA

## Abstract

Trajectory tracking is one of the key technologies for tractor automatic navigation. Its main purpose is to adjust the steering mechanism of the tractor to follow the planned trajectory. Thus, in this paper a trajectory tracking control system is designed for an agricultural tractor with the electric power steering mechanism. A DC brush motor is added on the steering column of the tractor and the hardware circuits for the steering controller are designed to control the front wheel angel. The three degrees of freedom model of the tractor is established, and a trajectory tracking control system is proposed including a fuzzy sliding mode controller and a steering angle tracking controller designed according to the internal mode control and minimized sensitivity theory. The effectiveness of the designed trajectory tracking control system is demonstrated by simulation analyses in reference to the planed trajectory.

## Introduction

Agriculture autonomous navigation is a key technology used in precision and intelligence agriculture, most especially in agricultural equipment. It has been applied to several agricultural implement in recent years, such as agricultural tractors, combines and sprayers [1–3]. Automatic navigation of tractor is an important component of precision agriculture. It plays a crucial role in the development of precision agriculture with the continuous research associated with its improvement. However, the diversity and complexity of farm working environment has been proposing a higher requirement for autonomous navigation of agricultural tractors in the operating process [4, 5]. The primary purpose of automatic tractor navigation is to control the moving trajectory of the tractor along the desired trajectory to be as accurate as human driving [6, 7]. To set the trajectory and calculate the error between the planned path and the current position of the tractor, curve generation method was presented to generate path of the whole vehicle and tracking control was designed according to the geometric method [8]. And the navigation controller is used to determine the expected steering angle for the guide wheel of tractors with a control algorithm according to the actual deviation between the tractor and the reference trajectory. As a research hotpot in this field, a variety of trajectory tracking and steering control algorithms have been proposed by many researchers and experts at home and abroad [9, 10].

Generally, the trajectory tracking control of autonomous tractor navigation consists of two parts, longitudinal control and lateral control [11]. To be specific, the variety of longitudinal control is the velocity of the tractor that depends on the accelerator pedal or brake actuator, and the steering angle is seen as a control target of lateral control for automatic driving tractors [12]. By contrast, the control of lateral position is more important than the control of longitudinal velocity because the performance of trajectory tracking relies on the lateral displacement to a great extent. On the other hand, a typical autonomous guidance system can be split into two broad categories, hardware and software [13]. The hardware part contains positioning devices, various sensors and actuators, such as a GPS or a BEIDOU, an angle sensor. And the program of the navigation system and control algorithm are regarded as software part.

At present, many researchers have been worked on RTK-GPS based guidance systems to offer better accuracy for autonomous tractors [14]. For instance, to reduce the lateral deviations of path tracking for an autonomous guidance tractor in paddy fields, Han et al. [15] developed an improved path tracking controller and built a test platform for a real tractor equipped with an RTK-GPS and IMU system. To meet the real-time vision navigation of the smart tractor in a complex agricultural field environment, Lu et al. [16] proposed an improved anti-noise morphology vision navigation algorithm and a camera and RTK-GPS were adopted on the tractor. However, the positioning accuracy of GPS is vulnerable to obstacles and electromagnetic interference around the farmland, and RTK consumes relatively large amounts of power, which makes it difficult to ensure the continuity of the navigation system. Automatic navigation steering devices are generally divided into electro-hydraulic drive type and motor drive type. The electro-hydraulic automatic steering system is usually installed in parallel with electro-hydraulic reversing valve and proportional valve on the original steering system to form an electro-hydraulic automatic steering circuit to realize automatic steering [17–20]. However, hydraulic leakage is inevitable for tractor automatic hydraulic steering system, and with the rapid popularization and promotion of servo control technology, stepper motor and servo motor have been more and more widely used in the automatic navigation and steering system of agricultural machinery [21, 22]. A DC brush motor was innovatively installed on the steering column to realize the steering control as low-cost, more precise, and easier maintenance by Yin et al. [23] in 2019.

Over the years, several control algorithms were proposed by researchers to obtain superior control performance. Among them, PID control methods have been used widely in the early days. The trajectory tracking control of unmanned agricultural vehicles was researched by Eski et al. [24] based on a neural network PID control system. In Ref. [25], a self-tuning fuzzy PID following controller for an agricultural tractor was designed. Also, the sliding mode control method was used gradually in agricultural machinery in recent years, such as Taghia et al. [26] presented a novel sliding mode controller with a nonlinear disturbance observer based on the kinematic and dynamic model to follow accurately a reference path. The adaptive neural network sliding mode control method was also adopted in the camera-based tractor-trailer trajectory tracking system [27]. In addition to the methods mentioned above, other contributions are also available, such as Zhao et al. [28] used force sensor to eliminate the uncertainty of the parameters of the trailers and developed a trajectory tracking controller based on backstepping techniques. To improve the stability and trajectory tracking performance, a linear dynamic model for the tractor was established and a linear quadratic regulator was designed by setting sideslip angle and lateral acceleration as optimization objectives [29]. However, the above-mentioned methods all consider the ideal model of the tractor. The soft soil of the farmland will affect the tracking accuracy of the self-driving agricultural machinery [30]. Accurate sideslip angle estimation is crucial for vehicle stability control [31]. To track the arbitrarily curved path in the presence of wheel slippage, Chen et al. [32] developed a path tracking controller based on second order sliding mode control and finite time disturbance observer technique. In particular, some control methods for road vehicles in the case of tire sideslip can be referenced to counter this problem [33–35].

From the above analysis, it can be seen that GPS positioning and hydraulic automatic steering system are adopted for most of the tractor navigation systems. In this paper, according to the motor power steering system proposed in the previous work [23], a navigation controller with BEIDOU positioning and gyroscope, and motor automatic steering system are constructed. In the proposed scheme, the controller gains are updated based on fuzzy rules to make up for the lack of the sliding mode control technology with jitter, and the trajectory tracking accuracy of the unmanned agricultural tractor is improved. For clearer illustrations, the paper contains 7 sections: the whole structure of the trajectory tracking system is shown in Section 2. In Section 3, a three degrees of freedom (DOF) tractor model and steering system model are established. The hardware circuits of the steering control system based on aided steering motor are designed in Section 4. The control strategies are proposed in Section 5. Simulation results and analysis are given in Section 6. Finally, some conclusions are presented in Section 7.

## Structure of trajectory tracking system

### Overall system configuration

**Fig 1:**
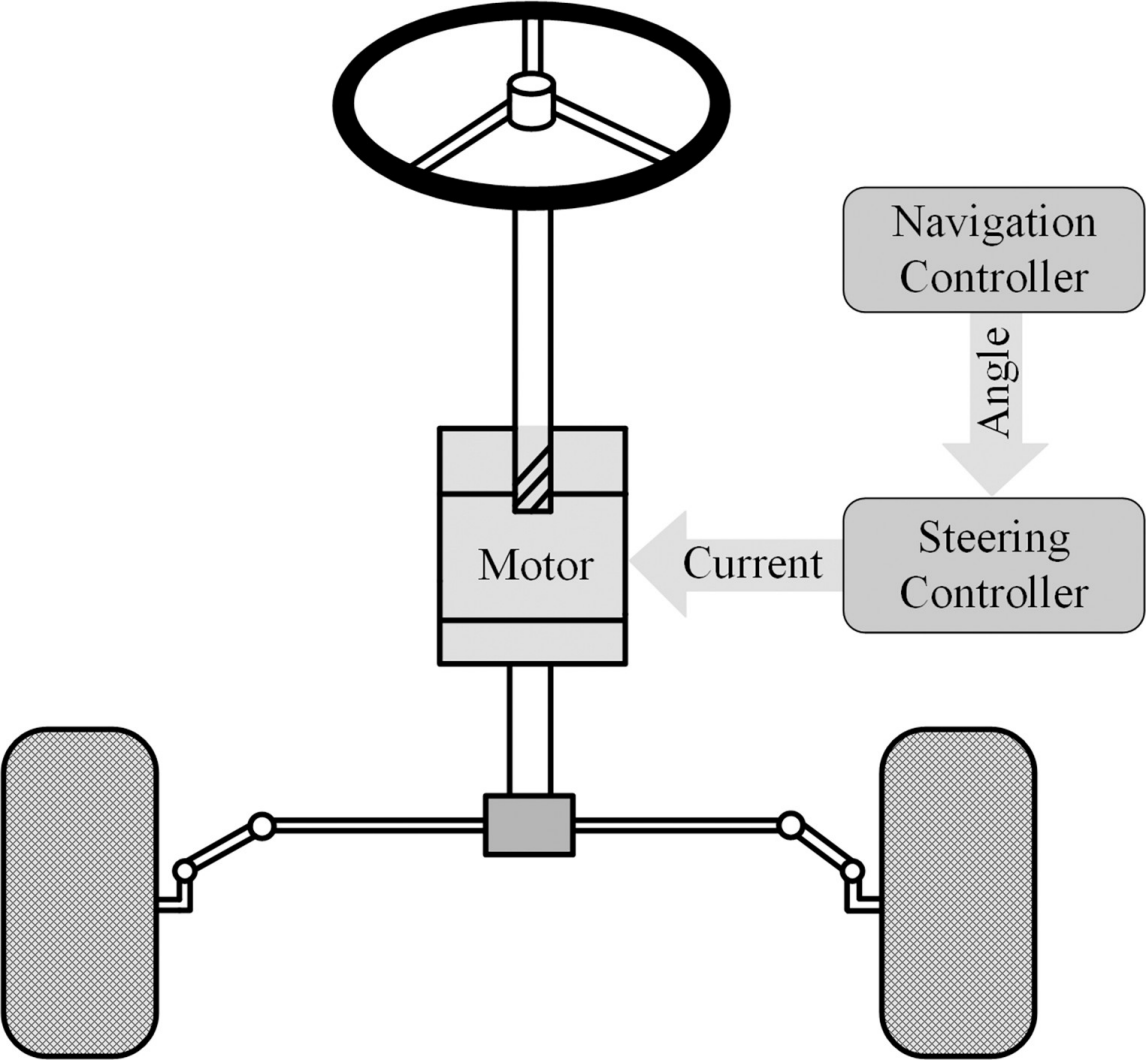
Trajectory tracking control system structure.

The whole structure of the tractor trajectory tracking control system is shown in Fig 1 which contains the navigation controller and steering controller. The navigation controller is used to process information from the BEIDOU antenna and gyroscope, and provide the desired steering angle for the steering controller. The steering controller is designed to control the steering motor according to the information received from the navigation controller and other sensors.

### Construction of trajectory tracking control system

**Fig 2:**
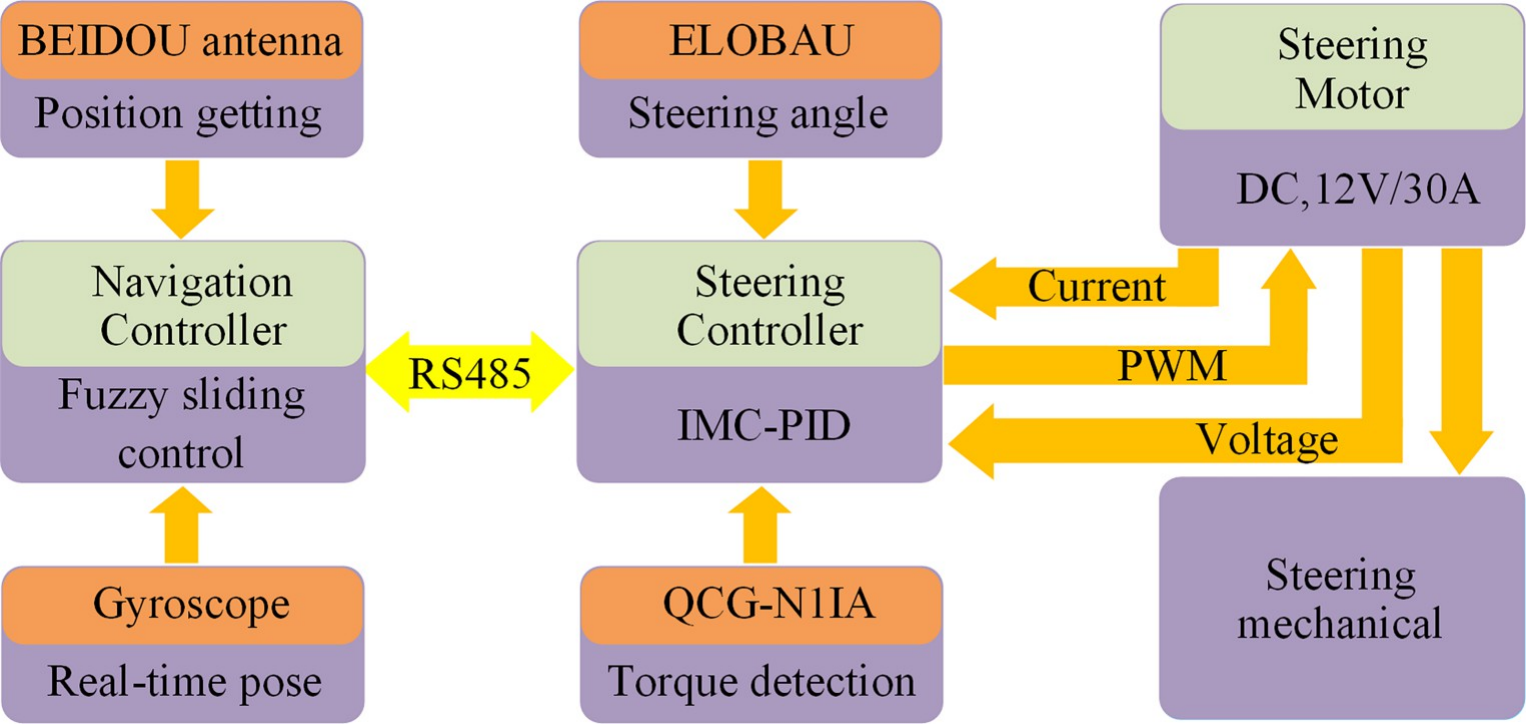
Hardware structure of the trajectory following the system.

The hardware structure of the path following system is shown in Fig 2. In the system, the BEIDOU double antenna and Gyroscope are installed on the tractor to obtain absolute position information. The navigation controller can calculate lateral error and steering angle according to the data of absolute position. The steering angle and torque are collected by ELOBAU and QCG-N1IA sensors respectively. The steering angle is transferred to the steering controller through the RS485 interface. After processing the above information, the steering motor is adjusted through PWM control mode.

## System model

### Model of the tractor

**Fig 3:**
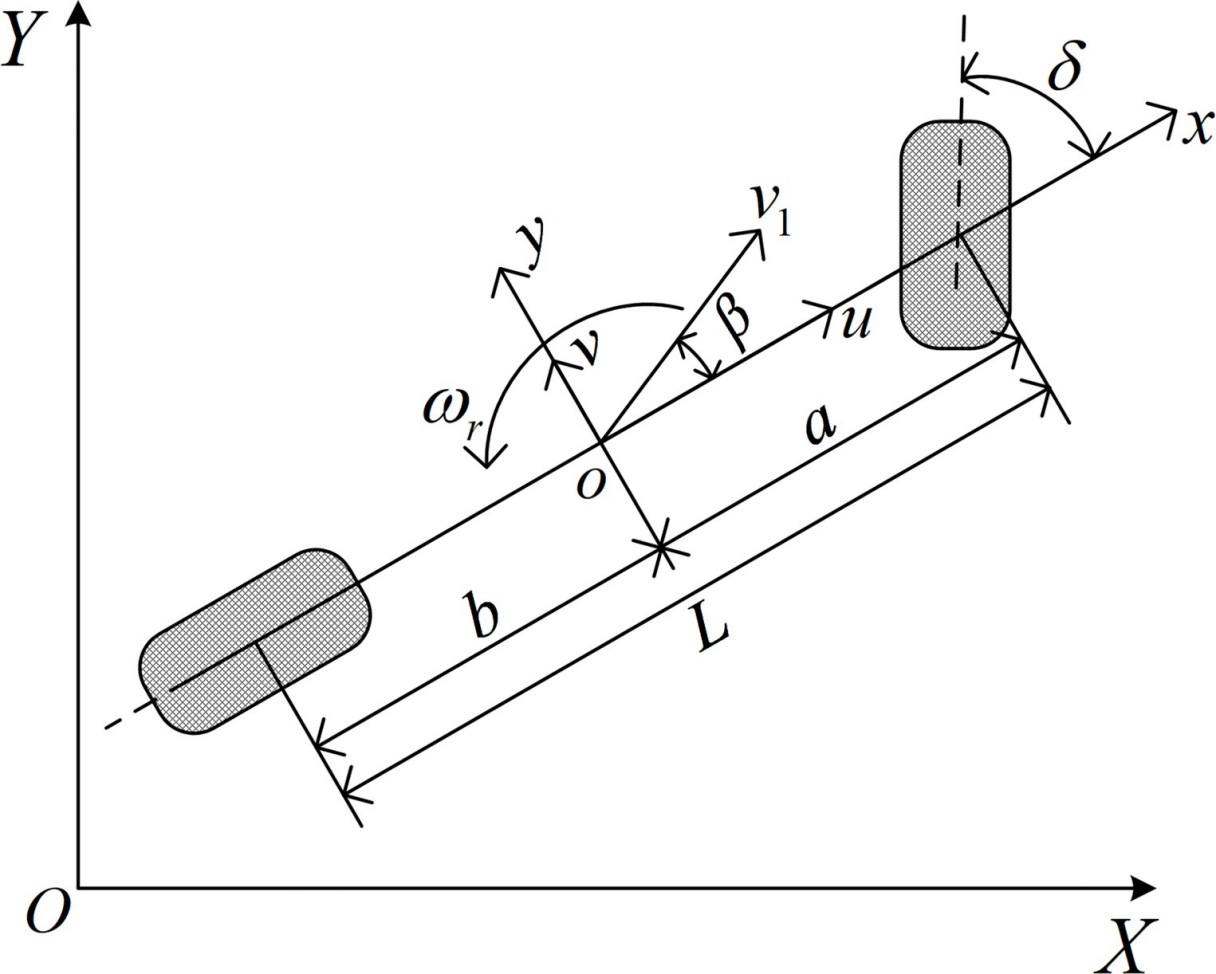
Three DOF model of a tractor.

The operating environment of tractor is very complex compared to that of a road or rail vehicle because it operates in grasslands, woodlands, and wetlands, etc. [36]. Therefore, it is difficult to establish an accurate mathematical model for a tractor in motion status. To solve this problem, multiple DOF model haven been developed by many researchers. However, such models are difficult to control. But, as we all know, tractors travel in a straight line usually. What’s more, the fact that tractor changes speed while driving is taken into consideration. Therefore, a three DOF model is developed based on the literature [37], with the heading angle, lateral displacement, and longitudinal velocity as shown in Fig 3.

According to Newton’s second law, the kinematic differential equation is established as follows.

$$\left\{ \begin{array}{c} \left(k_{1}+k_{2})\beta +\frac{ak_{1}+bk_{2}}{u}\omega_{r}-k_{1}\delta =m(\dot{v}+u\omega_{r}\right)\\ (ak_{1}+bk_{2})\beta +\frac{a^{2}k_{1}+b^{2}k_{2}}{u}\omega_{r}-ak_{1}\delta =I_{z}{\dot{\omega }}_{r} \end{array}\right.$$ (1)

where, *k*_1_ and *k*_2_ are cornering stiffness of the front and rear wheels, respectively; *β* = *v*/*u* is the angle of deviation from the center of mass; *a* and *b* are the distances between the front and rear axes to the center of mass, respectively; *ω*_*r*_ is the yaw angular velocity; *δ* represents the angle of the front wheel; *m* is the mass of tractor; *v* and *u* are the lateral and longitudinal velocity of the tractor, respectively.

In Fig 3, (*X*, *Y*) and (*x*, *y*) are used to demonstrate the geodetic and vehicular coordinates, respectively, which have the following relationship [38].

$$\left\{ \begin{array}{l} \dot{x}(t)=u\;\cos\;\theta (t)-v\;\sin\;\theta (t)\\ \dot{y}(t)=u\;\sin\;\theta (t)+v\;\cos\;\theta (t) \end{array}\right.$$ (2)

where *θ* is heading angle and $\dot{\theta }=\omega_{r}.\;\dot{x}(t)$ and $\dot{y}(t)$ denote the velocity components of the tractor in geodetic coordinates (*X*, *Y*), respectively.

The system state of the tractor can be defined as $x={\left[x_{1},x_{2},x_{3},x_{4}\right]}^{T}={\left[y,\dot{y},\theta ,\dot{\theta }\right]}^{T}\in R^{4},$ *U* is regarded as control input. The state equation can be transferred as following.

$$\begin{array}{l} {\dot{x}}_{1}=x_{2}\\ {\dot{x}}_{2}=f_{1}(x)+g_{1}U+d_{1}\\ {\dot{x}}_{3}=x_{4}\\ {\dot{x}}_{4}=f_{2}(x)+g_{2}U+d_{2} \end{array}$$ (3)

where,

$$\left\{ \begin{array}{l} f_{1}=m_{11}x_{2}-m_{11}x_{3}u+(m_{12}+u)x_{4}\\ g_{1}=-\frac{k_{1}}{m}\\ f_{2}=m_{21}x_{2}-m_{21}x_{3}u+m_{22}x_{4}\\ g_{2}=-\frac{ak_{1}}{I_{z}} \end{array}\right.$$ (4)

$m_{11}=\frac{k_{1}+k_{2}}{mu},m_{12}=\frac{ak_{1}-bk_{2}}{mu}-u,b_{11}=-\frac{k_{1}}{m},m_{21}=\frac{ak_{1}-bk_{2}}{I_{z}u},m_{22}=\frac{a^{2}k_{1}+b^{2}k_{2}}{I_{z}u},b_{21}=-\frac{ak_{1}}{I_{z}},$ *d*_1_ and *d*_2_ are unknown disturbances.

### Model of the steering system

**Fig 4:**
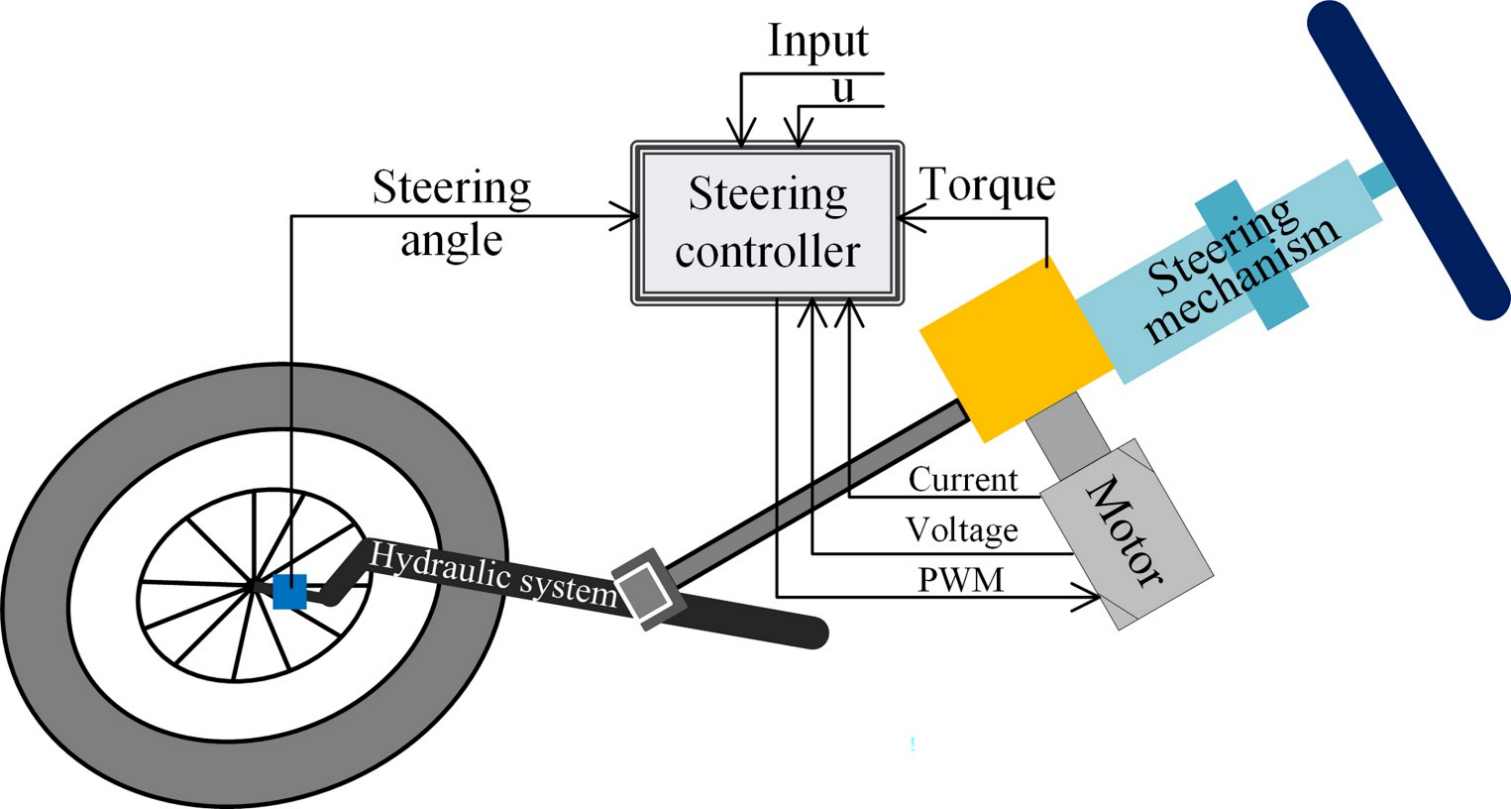
Steering system model diagram.

As designed in Fig 4, the steering system consists of a mechanical steering mechanism, a hydraulic steering system and an electric power steering system. A DC brush motor is used as the actuator for the automatic steering system. The models of the mechanical steering mechanism include the steering column model, the steering shaft model and the gear model. The model of the hydraulic steering system includes a model of the hydraulic cylinder, a model of the steering pump and a model of the valve. The model of the electric power steering system is mainly composed of a PWM power amplifier model and a power motor model. The steering system is also modelled with the assumptions of frictionless and hydraulic leakage free and rigid coupling. Thus, *u*(*s*) is used as the input to the control system and *δ*(*s*) is used as the output of the control system, and the following transfer function is obtained.


$$G(s)=\frac{\delta (s)}{u(s)}=\frac{K}{s(T_{1}s+1)(T_{2}s+1)}e^{-\theta s}$$ (5)

## Steering tracking control system design

### Overall structure of the steering control circuit

**Fig 5:**
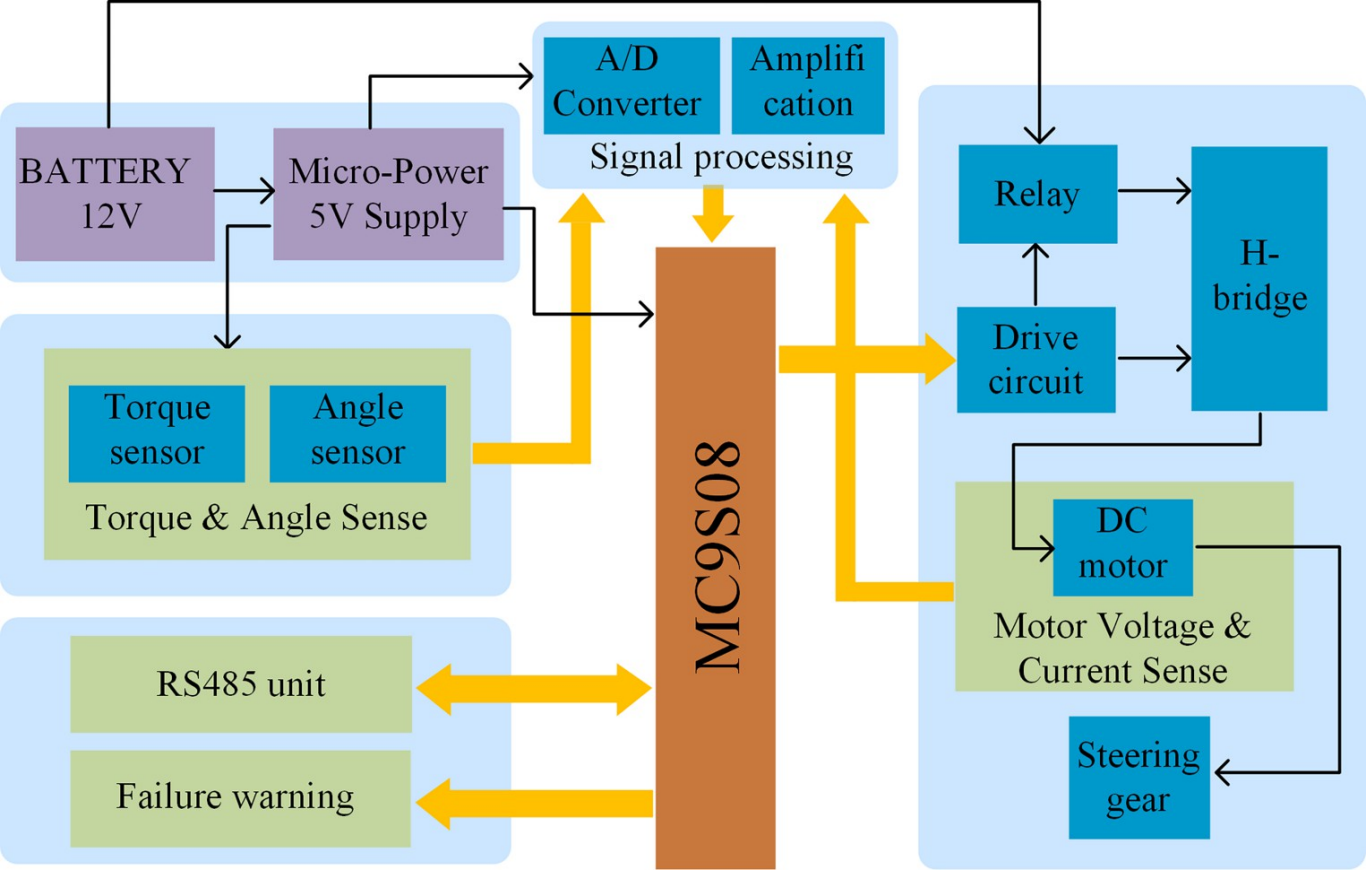
Block diagram of the control circuit.

The control circuits for the steering system based on assisted motor mainly include four parts: microcontroller, sensor acquisition circuit, drive circuit, and power supply circuit. The microcontroller is the control core of the steering system, and all detected signals are finally sent to the microcontroller for data analysis and comparison. The circuits of signal acquisition are mainly used for collecting torque signal, DC motor current and voltage signal. The DC motor is the actuator of the steering system, which is controlled by the H-bridge to realize steering. The power supply circuits provide electric energy for the whole steering system, including a 12V DC power supply and a 5V DC power supply. The overall block diagram of the control circuit is shown in Fig 5.

The microcontroller plays a key role in the stability, reliability and security of the whole steering control system. An 8-bit Freescale MC9S08 single-chip microcomputer with strong anti-interference and adaptation is chosen as a microcontroller in this study. Its operating temperature is between -40°C and +125°C and it is lower cost. Thus, it can meet the demand for steering operation on the tractor completely.

### Torque detection circuit

**Fig 6:**
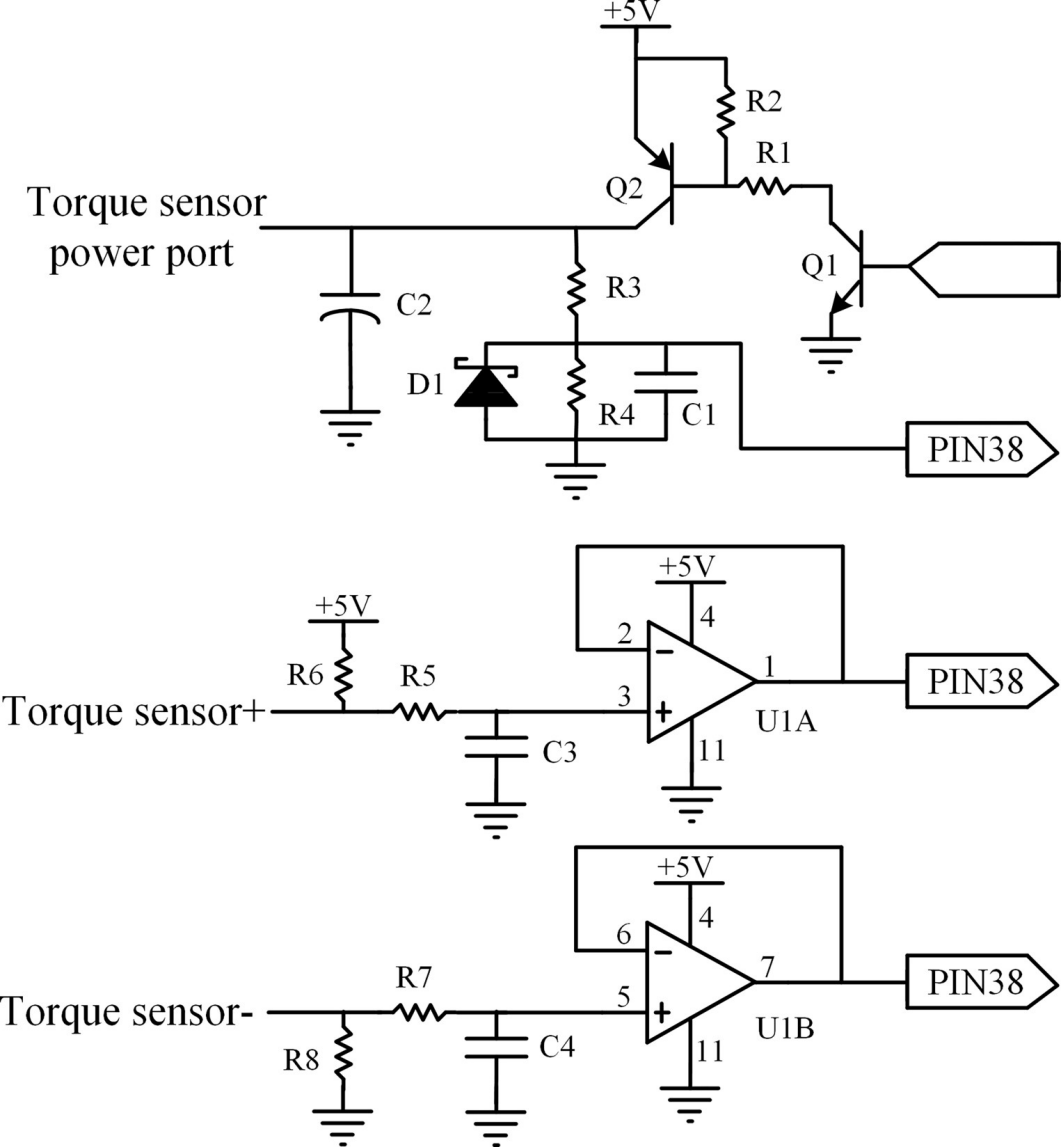
Schematic of the torque detection circuit.

In order to monitor the rotation direction of the motor, and at the same time check for any manual driving intervention, a potentiometer type torque sensor is set up on the steering shaft of the tractor. The voltages of the two input ports of the sensor are 5V (VCC) and 0V (GND), respectively, and the two output ports are the main torque signal and associate torque signal, respectively. A double loop output circuit is designed in the design of torque detection, which compares the associate torque signal with the main torque signal and estimates the state of the main torque signal to ensure the accuracy of the output signal of the torque sensor. The steering wheel is turned right when voltage signals of main torque more than 2.5V, otherwise the opposite. The value of output voltages is 2.5V if the steering wheel locates in the middle of the steering shaft, which means that the tractor is moving along a straight line. The schematic of the torque detection circuit is illustrated in Fig 6.

To reduce the energy consumption of the controller, two transistors are selected to control the +5V DC power for the torque sensor by applying their switch function, NPN and PNP type, respectively. The base of Q1 is controlled by a microcontroller, Q2 and Q1 are opened if the base of Q1 is high level, and then +5V DC is carried to the positive pole of the torque sensor. To ensure output voltage and input voltage are similar to the power supply voltage and avoid signal attenuation in the next circuit, a voltage following circuit is designed by using an LM324 operational amplifier.

### Relay control circuit

**Fig 7:**
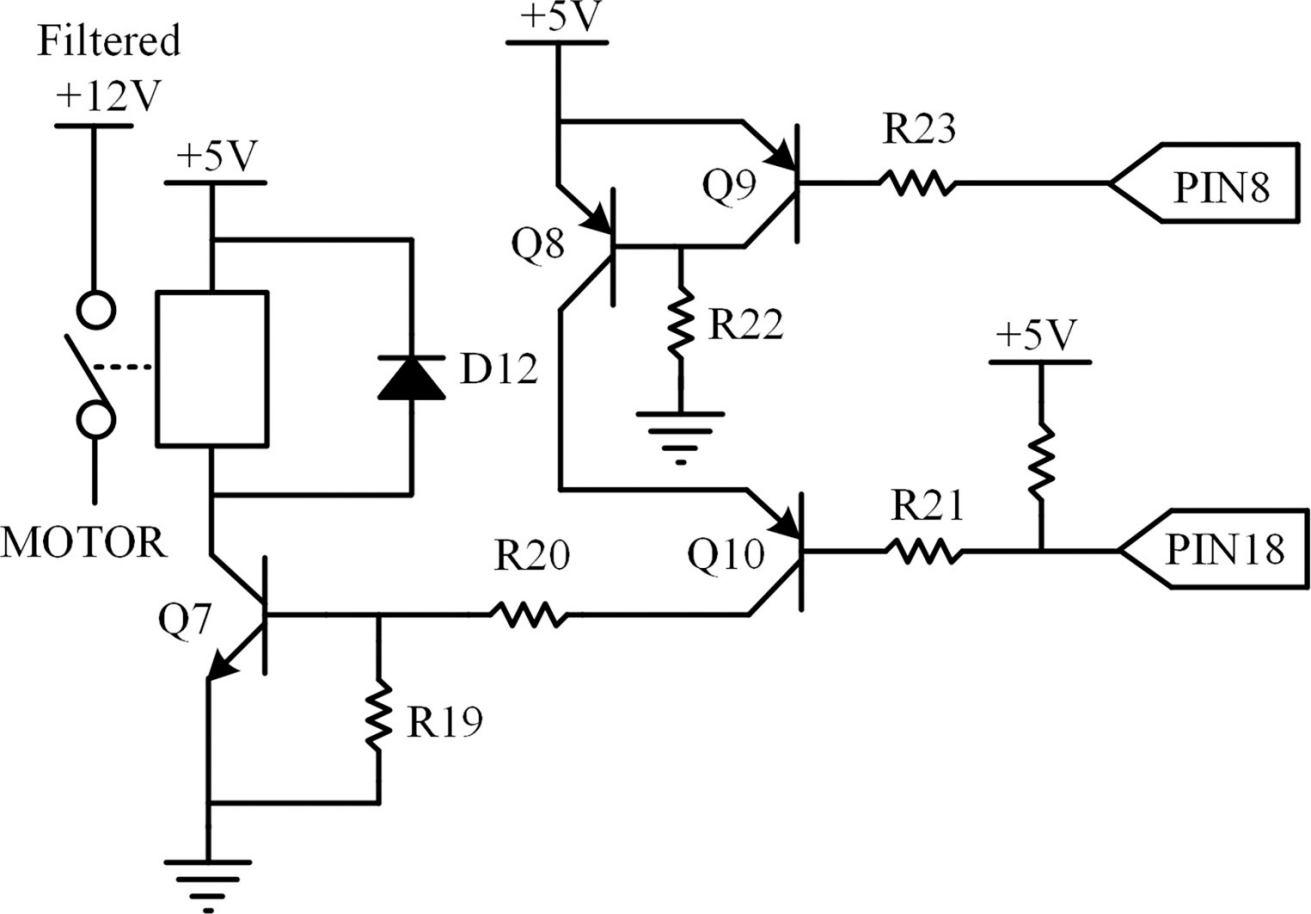
Schematic of the relay control circuit.

The relay is adopted to control the connection between the driving circuit and H-bridge when the system is not working and shuts off the power supply in the event of a failure to protect the ECU. The relay control circuit consists of a relay and four transistors. The Q9 is opened and Q8 is closed if the reset signal is low level, and so the Q10 is closed. Otherwise, the Q8, Q10, Q7 and relay are opened when the control signal is low level. As a result, the conflict between the reset circuit and control circuit can be avoided in operating. The schematic of the relay control is illustrated in Fig 7.

## Current control circuit

**Fig 8:**
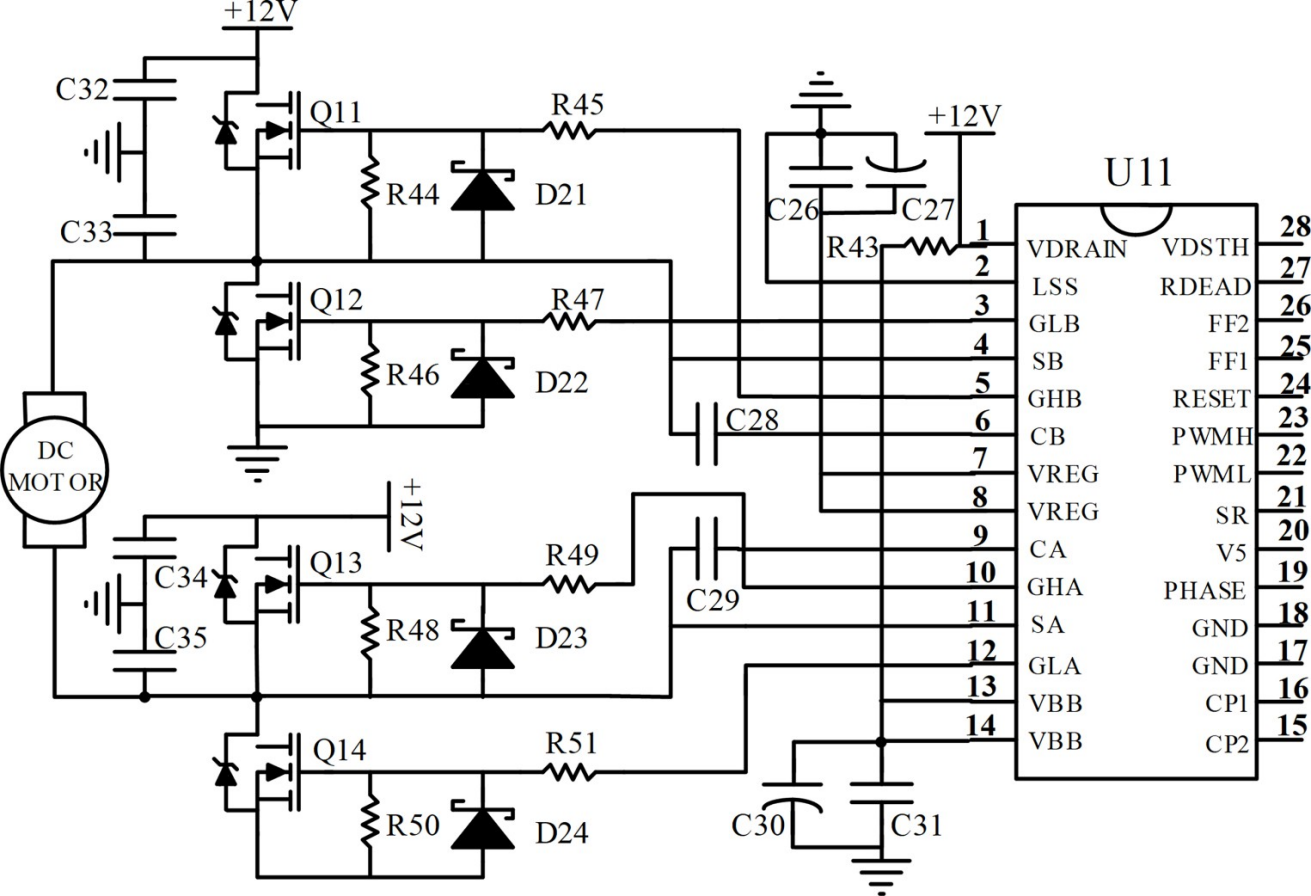
Schematic of current control.

**Fig 9:**
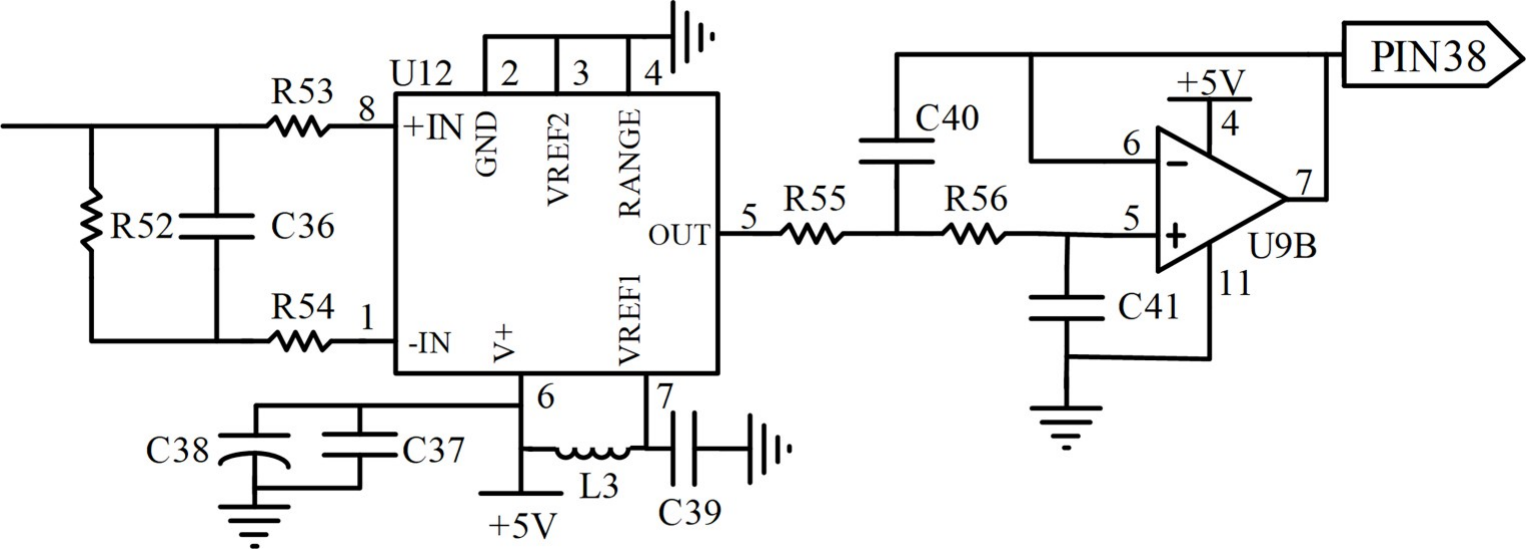
Schematic of current sensing.

Brush motor is extensively used because of its simple structure and higher accuracy of control. So, in this study a brush DC motor is selected as the steering motor of the tractor. Its rated current is 30A and the rated voltage is 12V. In order to control the velocity and rotation of the motor by PWM mode to follow the desired wheel angle, the current control circuit and sensing circuit are designed, the produced circuits are demonstrated in Figs 8 and 9 respectively. The designed current control circuits include an H-bridge MOSFET driver A3941 and four IRF3205 MOSFETs. For the first group, power tube Q11 and Q14 control the clockwise and speed of the motor. As for the second group, power tube Q12 and Q13 control the anti-clockwise and speed of the motor. And the H-bridge MOSFET driver is controlled by the microcontroller.

## Control strategies design

**Fig 10:**
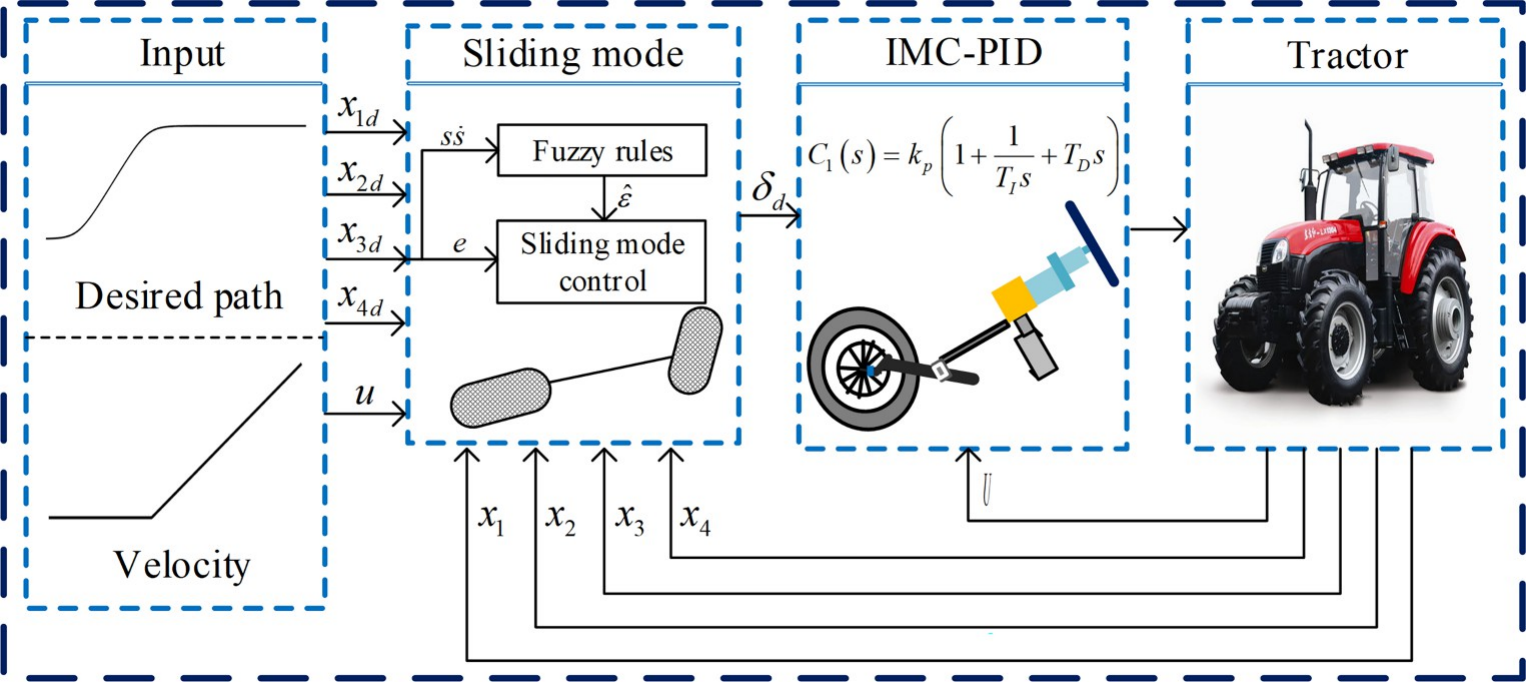
Overall structure of the control system.

The agricultural tractor is a relatively complex control object, and its motion characteristics are closely related to ground conditions, steering mechanism and various systematic errors which are difficult to obtain in tractor motion. The sliding mode controller has the advantage of fast response and insensitivity to parameter changes and perturbations [39]. However, for known mathematical models, the sliding mode controller can make the system track the desired instruction, but for models with large switching gains, it tends to exacerbate system jitter. The outstanding advantage of fuzzy logic is that it is relatively easy to incorporate human control experience into the controller through fuzzy rules, thereby higher precision control can be obtained [40]. A trajectory tracking control system based on fuzzy sliding mode control algorithm is proposed in Fig 10. The trajectory tracking control system consist of a fuzzy sliding mode controller and IMC-PID controller. In the operation, the lateral deviation *e* is determined by the BEIDOU antenna and the Gyroscope. Both the sliding mode controller and the fuzzy rules are designed based on the lateral deviation. The angle tracking controller is used to adjust the steering motor according to *δ* according to the measured value from the angle sensor.

### Fuzzy sliding mode control law design

The basic design steps of a sliding mode control law consist of two parts: switching function design and approximation law design. The lateral deviation *e* is defined as

$$e=x_{i}-x_{id}\;(i=1,3)$$ (6)

where *x*_*id*_ is the desired trajectory information.

The switch function associated with the deviation is designed as

$$s_{\kappa }=\lambda_{\kappa }e_{\kappa }+{\dot{e}}_{\kappa }$$ (7)

where *κ* = 1,2, *λ*_*κ*_>0.

The saturation function with subsystem is defined as

$$z=z_{g}\;\sin\left(s_{\kappa }\right)\;\kappa =2$$ (8)

Coupling sliding mode surface is designed as

$$S=s_{\kappa }-\lambda_{\kappa }z\;\kappa =1$$ (9)

The exponential approach law is chosen as the sliding mode control law.

$$\dot{S}=-kS-\varepsilon \;\mathrm{sgn}\left(S\right)={\dot{s}}_{1}-\lambda_{1}\dot{z}$$ (10)

where *ε*>0, and the convergence rate of the system is determined by *ε*.

According to Eq (3), Eq (8), Eq (9) and Eq (10), the control law can be obtained

$$U=U_{eq}+U_{sw}$$ (11)

where, *U*_*eq*_ is the equivalent input and *U*_*sw*_ is switch control input, they are expressed as follows.

$$\left\{ \begin{array}{l} U_{eq}=-\frac{1}{\zeta }\left\{\begin{array}{c} \lambda_{1}{\dot{e}}_{1}+f_{1}(x)-{\dot{x}}_{2d}\\ -\xi [\lambda_{2}{\dot{e}}_{2}-f_{2}(x)+{\dot{x}}_{4d}] \end{array}\right\}\\ U_{sw}=-\frac{1}{\zeta }[kS+\varepsilon \;\mathrm{sgn}\left(S\right)] \end{array}\right.$$ (12)

where $\zeta =g_{1}+g_{2}\xi ,\xi =\lambda_{1}z_{g}\;\cos\left(s_{2}\right)$.

It can be seen from Eq (12) that the jitter of sliding mode control is directly caused by the discontinuous $\dot{S}$. Since the symbol function *sgn*(*S*) cannot be changed, the system jitter can be attenuated by the coefficient *ε* to improve the performance of the trajectory tracking system. From Eq (10), it can be seen that *ε*(*t*) is the main cause of jitter. *ε*(*t*) is used to compensate the disturbance *d*(*t*) to ensure the existing condition of sliding mode is satisfied. However, *d*(*t*) is time-varying during tractor operation, and *ε*(*t*) should also be time-varying to reduce chattering. Therefore, fuzzy rules are adopted to realize the change of *ε*(*t*) according to the human experience.

First, $S\dot{S}$ and *Δε*(*t*) are defined as the input and output of the fuzzy controller respectively.

$$S\dot{S}=\{\begin{array}{ccccccc} NB & NM & NS & ZO & PS & PM & PB \end{array}\},$$

$$\Delta \varepsilon (t)=\{\begin{array}{ccccccc} NB & NM & NS & ZO & PS & PM & PB \end{array}\}.$$

where *NB* is negative big, *NM* is the negative middle, *NS* is negative small, *ZO* is zero, *PS* is positive small, *PM* is the positive middle, *PB* is positive big.

The upper bound of $\hat{\varepsilon }(t)$ is estimated by the integral method as follows.

$$\hat{\varepsilon }(t)=G\int_{0}^{t}\Delta \varepsilon (t)dt$$ (13)

where *G* is the coefficient of proportionality, empirically determined.

By combining Eq (10) and Eq (12), the fuzzy sliding mode control law can be obtained as follows.


$$\left\{ \begin{array}{l} U_{eq}=-\frac{1}{\zeta }\left\{\begin{array}{c} \lambda_{1}{\dot{e}}_{1}+f_{1}(x)-{\dot{x}}_{2d}\\ -\xi [\lambda_{2}{\dot{e}}_{2}-f_{2}(x)+{\dot{x}}_{4d}] \end{array}\right\}\\ U_{sw}=-\frac{1}{\zeta }[kS+\hat{\varepsilon }\;\mathrm{sgn}\left(S\right)] \end{array}\right.$$ (14)

To prevent $\hat{\varepsilon }(t)$ from being too large and causing the steering input signal *δ* to be too large or $\hat{\varepsilon }(t)$ less than 0, a mapping adaptive algorithm is adopted to correct $\hat{\varepsilon }(t)$ timely [41].


$$\dot{\hat{\varepsilon }}(t)=Proj_{\hat{\varepsilon }}(G\int_{0}^{t}\Delta \varepsilon (t)dt)$$ (15)

$$Proj_{\hat{\varepsilon }}(•)=\left\{ \begin{array}{l} \begin{array}{cccc} 0 & if\;\hat{\varepsilon }\geq \varepsilon_{max} & and & •>0 \end{array}\\ \begin{array}{cccc} 0 & if\;\hat{\varepsilon }\leq \varepsilon_{min} & and & •<0 \end{array}\\ \begin{array}{cc} • & otherwise \end{array} \end{array}\right.$$ (16)

In order to prove that the proposed sliding mode control method can make the tractor coincide with the desired trajectory, which is the tracking error attenuates to zero, $\underset{t\to \infty }{lim}{\left[y,\dot{y},\theta ,\dot{\theta }\right]}^{T}={\left[y_{d},0,\theta_{d},0\right]}^{T}$.

In order to prove the sliding mode surface constructed according to Eqs (6)–(9) is asymptotically stable with the controller designed by Eq (12), the Lyapunov function is selected as

$$V=\frac{1}{2}S^{2}$$ (17)

$$\dot{V}=S\dot{S}$$ (18)

Combining Eq (18) with Eqs (9) and (10) yields the following

$$\dot{V}=-kS^{2}-\varepsilon \mathrm{sgn}\left(S\right)\leq -kS^{2}-\varepsilon |S|\leq 0$$ (19)

Integrating both sides of Eq (19), we can get the following inequation

$$V(t)-V(0)=-\int_{0}^{t}\left(kS^{2}+\varepsilon S\right)d\tau \leq 0$$ (20)

As can be seen from Eq (20) that

$$-\int_{0}^{t}\left(kS^{2}+\varepsilon S\right)d\tau \leq V(0)<\infty$$ (21)

It can be observed from Eqs (19) and (21) that $S\in L_{\infty },\dot{S}\in L_{\infty }$. According to Barbalat’s Lemma, $\underset{t\to \infty }{lim}S=0$, the sliding mode surface of the system is asymptotically stable.

What’s more, to prove the sliding mode surface of the subsystem constructed according to Eqs (6)–(7) is asymptotically stable with the controller designed by Eq (12), intermediate variables $\tilde{z}$ is defined as

$$\tilde{z}=c_{g}\;\sin\left(s_{2}\right)$$ (22)

Eq (22) shows that $\tilde{z}\in L_{\infty }$. So the following equations can be got

$$S=s_{1}-z_{g}\tilde{z}$$ (23)

$$\dot{S}={\dot{s}}_{1}-z_{g}\dot{\tilde{z}}$$ (24)

Substituting Eq (7) into Eq (3), it can be got as

$${\dot{s}}_{1}=\lambda {\dot{e}}_{1}+f_{1}+g_{1}U-{\dot{x}}_{2d}$$ (25)

According to Eq (25), the right side of the Equation is bounded, which means ${\dot{s}}_{1}\in L_{\infty }$. Because $\dot{S}\in L_{\infty }$, it can be further concluded that $\dot{\tilde{z}}\in L_{\infty }$. Based on Eq (23), it follows from Barbalat’s Lemma that $\underset{t\to \infty }{lim}s_{1}=0$. Further, $\underset{t\to \infty }{lim}s_{2}=0$. In summary, the sliding mode surface *s*_*κ*_ is asymptotically stable. Which means $\underset{t\to \infty }{lim}{\left[y,\dot{y},\theta ,\dot{\theta }\right]}^{T}={\left[y_{d},0,\theta_{d},0\right]}^{T}$.

### Steering angle tracking control law design

**Fig 11:**
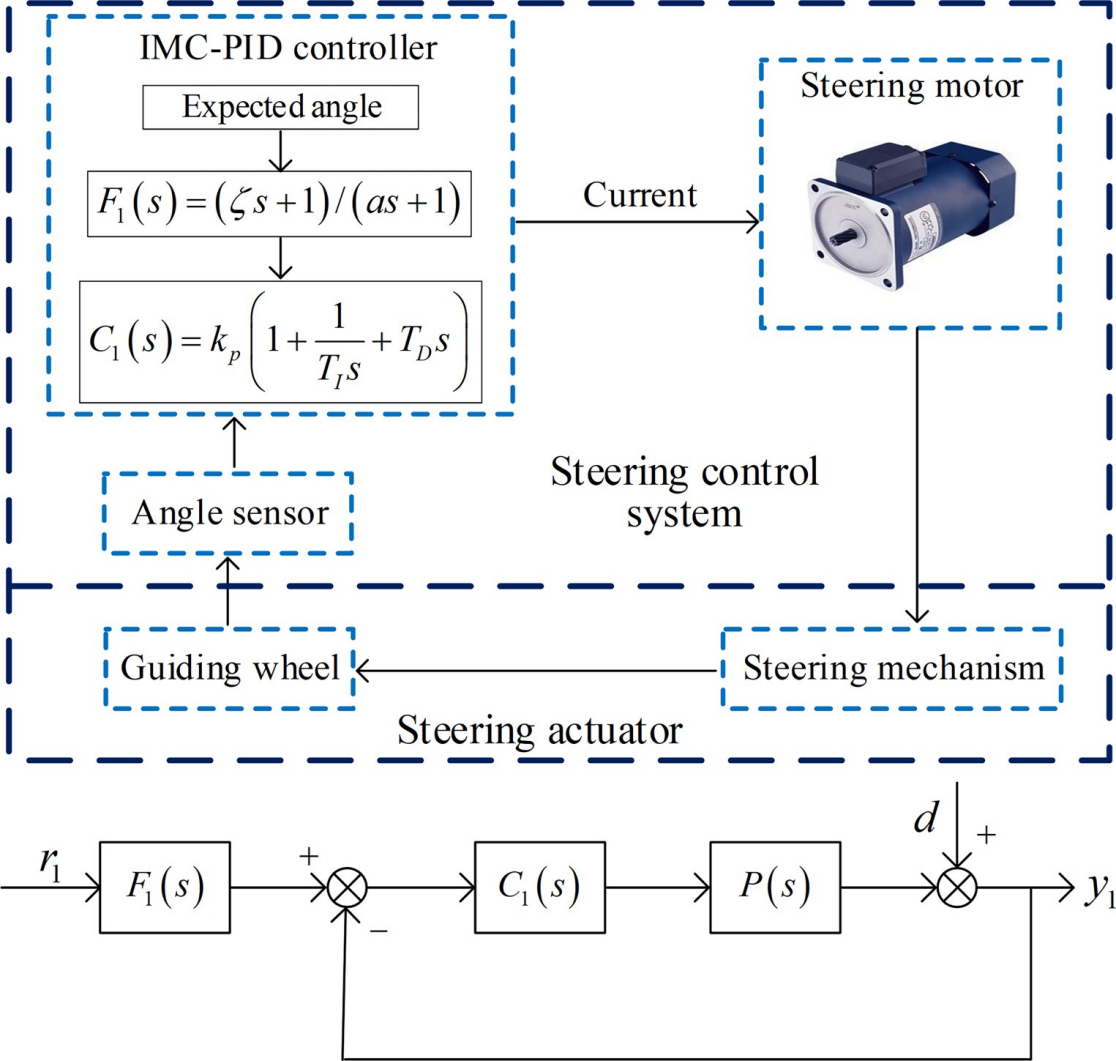
Structure diagram of the steering angle tracking control strategy. (a) Block diagram of steering system control, (b) System structure of steering control.

The structure diagram of the steering angle tracking control strategy is shown in Fig 11. According to the relationship between the internal mode control and unit feedback control, minimized sensitivity method is adopted to design the controller.


$$Q(s)=C_{1}(s)/(1+C_{1}(s)P(s))$$ (26)

For the unity feedback control structure adopted for the steering angle control, the sensitivity function of the closed loop between the process input and output for the load disturbance rejection can be obtained as

S(s)=1−Q(s)P(s) (27)

Define the optimal performance criteria be min ‖*W*(*s*)*S*(*s*)‖_∞_, where *W*(*s*) is the weighting function. Usually, in process control, *W*(*s*) can be selected as 1/*s*. According to the *H*_∞_ control theory and the well-know maximum modulus theorem, it can be got

$${\left\|W(s)S(s)\right\|}_{\infty }={\left\|W(s)(1-Q(s)P(s))\right\|}_{\infty }\geq |W(1/(\theta_{1}+\theta_{2}))|$$ (28)

Approximating the time delay by a first-order Taylor series *e*^−*θs*^ = 1−*θs*, we can obtain the approximated steering system as

$$P(s)=K(1-\theta s)/s(T_{1}s+1)(T_{2}+1)$$ (29)

Accordingly, the angle tracking controller *C*_1_(*s*) can be derived by the above design procedure, that is

$$C_{1}(s)=\frac{s(T_{1}s+1)(T_{2}s+1)(as+1)}{K[{\left(\zeta s+1\right)}^{4}-(1-\theta s)(as+1)]}$$ (30)

where, *a* = 4*ζ*+*θ*. Mathematical Maclaurin expansion series is utilized to copy out the controller *C*_1_(*s*) in Eq (30), letting *C*_1_(*s*) = *M*(*s*)/*s*, and the executable *C*_1_(*s*) can be obtained in form of PID by using Eq (30) and Eq (31),

$$C_{1}(s)=\frac{1}{s}\left[f(0)+f^{\prime }(0)s+\frac{f^{\prime \prime }(0)}{2!}s^{2}+\ldots \right]$$ (31)

The first three terms of the above Maclaurin expansion constitute exactly a standard PID controller in the form of

$$C_{1}(s)=k_{p}(1+\frac{1}{T_{I}s}+T_{D}s)$$ (32)

where $k_{p}=f^{\prime }(0),T_{I}=f^{\prime }(0)/f(0)$, and $T_{D}=f^{\prime \prime }(0)/2f^{\prime }(0)$.

In this work, a set point filer is selected to improve the steering angle tracking performance and reduce the overshoot, and the setpoint filer is designed *F*_1_(*s*) = (*ζs*+1)/(*as*+1).

## Simulation results

To verify the effectiveness of the designed fuzzy sliding mode controller and IMC-PID for agricultural tractor, the simulation model is established using Matlab in terms of three DOF model and control strategies in Section 5. The tractor parameters in the built model are shown in Table 1.

**Table 1:** The parameters of the tractor.

Parameters	Units	Values
*m*	kg	1818.2
*I*_*z*_	kg•m^2^	3885
*L*	m	3.048
*a*	m	1.463
*b*	m	1.585
*k*_1_	KN•rad^-1^	-62618
*k*_2_	KN•rad^-1^	-110185

**Fig 12:**
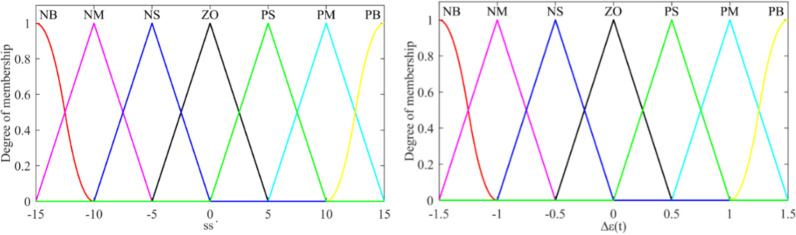
Membership function of fuzzy system. (a) Membership function of fuzzy input, (b) Membership function of fuzzy output.

The parameters of the steering system model are confirmed *K* = 13.5, *T*_1_ = 0.2, *T*_2_ = 0.3, *θ* = 0.15, and the adjustable parameter for the steering tracking controller is selected as *k*_*p*_ = 0.0966, *T*_*I*_ = 2.3757, *T*_*D*_ = 0.189, *ζ* = 0.5. The parameters of the ordinary sliding mode controller are as follows: *λ*_1_ = 5, *λ*_2_ = 13, *k* = 20, *ε* = 5, *z*_*g*_ = 0.05, and the membership function of the fuzzy sliding mode controller is shown in Fig 12.

Considering the velocity of the tractor is not constant, but changes with the actual road conditions. The initial velocity of tractor *u* is 5.5m/s, the velocity increases to 11m/s as the road flattens out. To show more clearly the superiority of the designed fuzzy sliding mode control system, the ordinary sliding mode controller, the modified sliding mode controller proposed by Zhao *et al*. [42] are introduced to compare with the fuzzy sliding mode controller, respectively, where using continuous function *s*/(|*s*|+*δ*) to replace *sgn*(*s*), including *δ* = 0.01 proposed by Zhao *et al*. and *G* = 10 for fuzzy sliding mode control.

**Fig 13:**
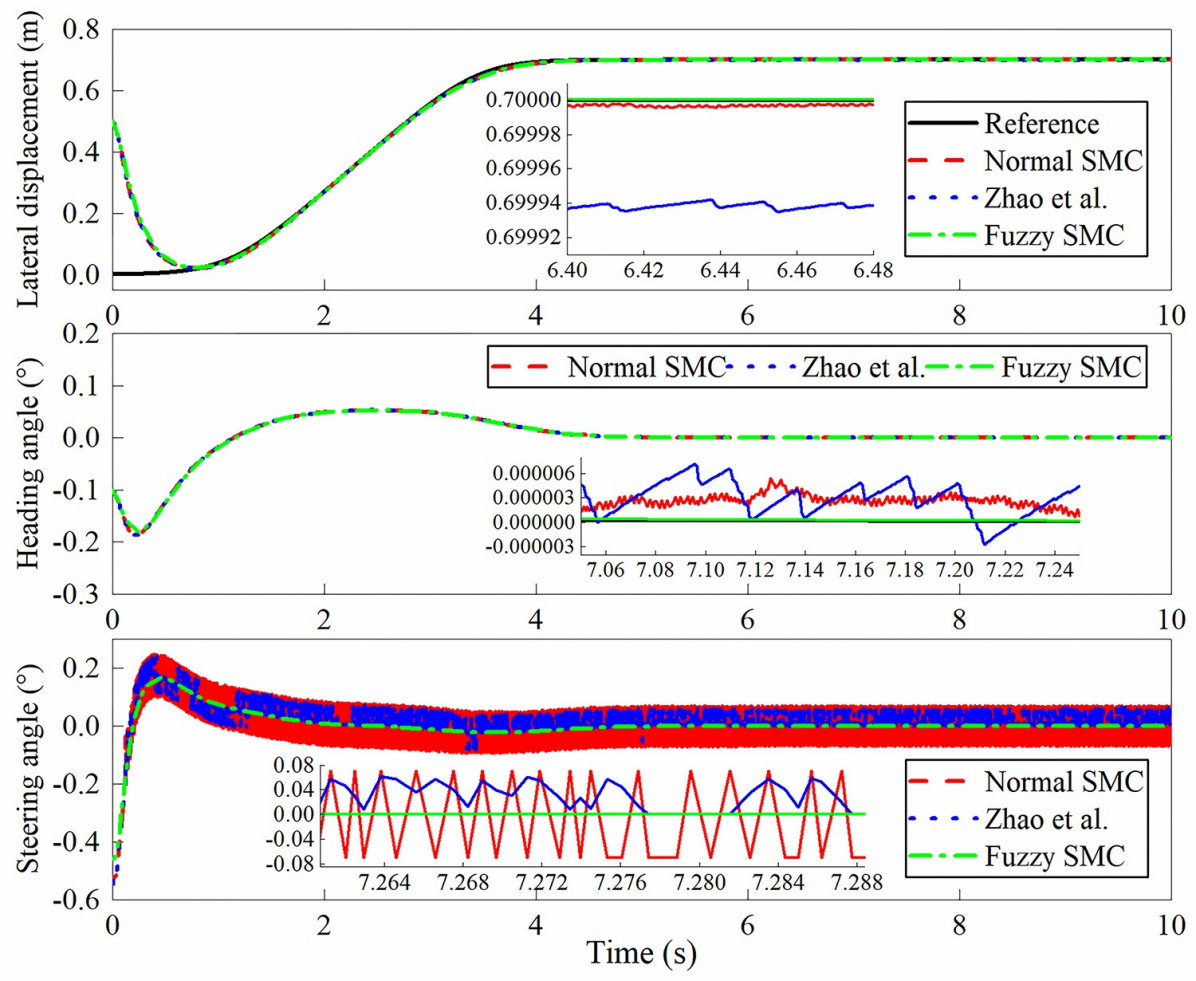
Lateral displacement, heading angle, steering angle.

**Fig 14:**
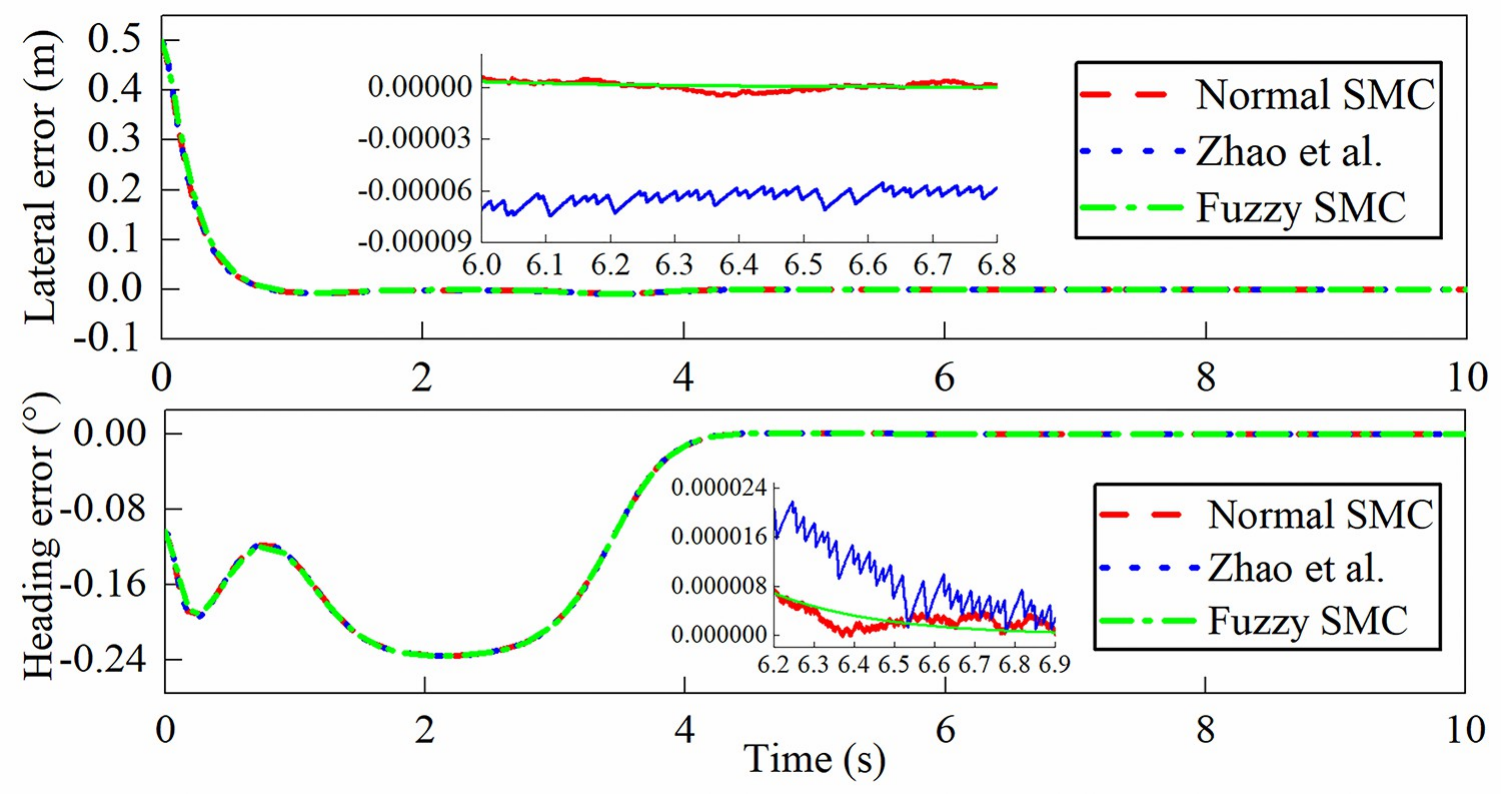
Lateral error, heading error.

**Fig 15:**
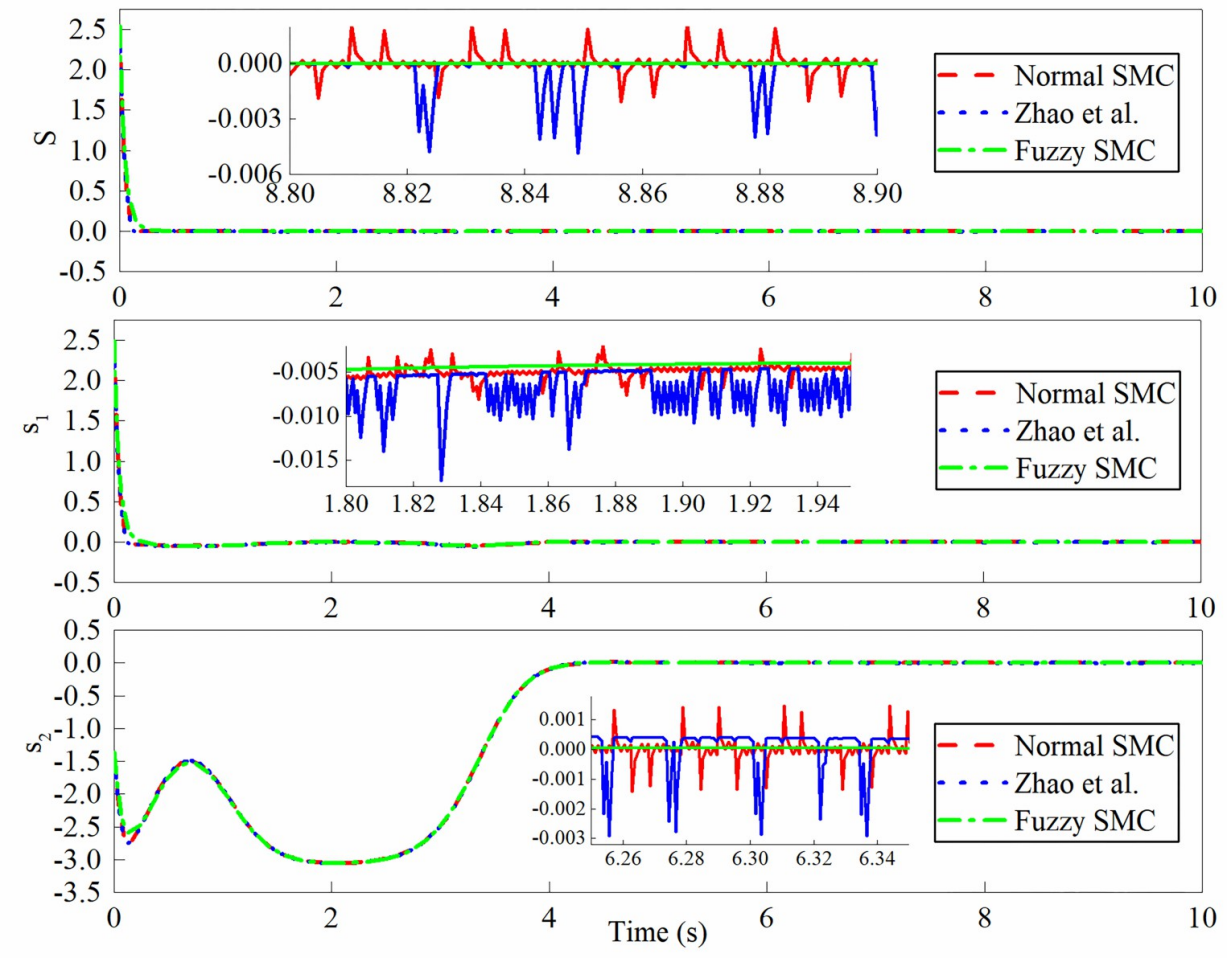
Sliding mode surfaces.

**Fig 16:**
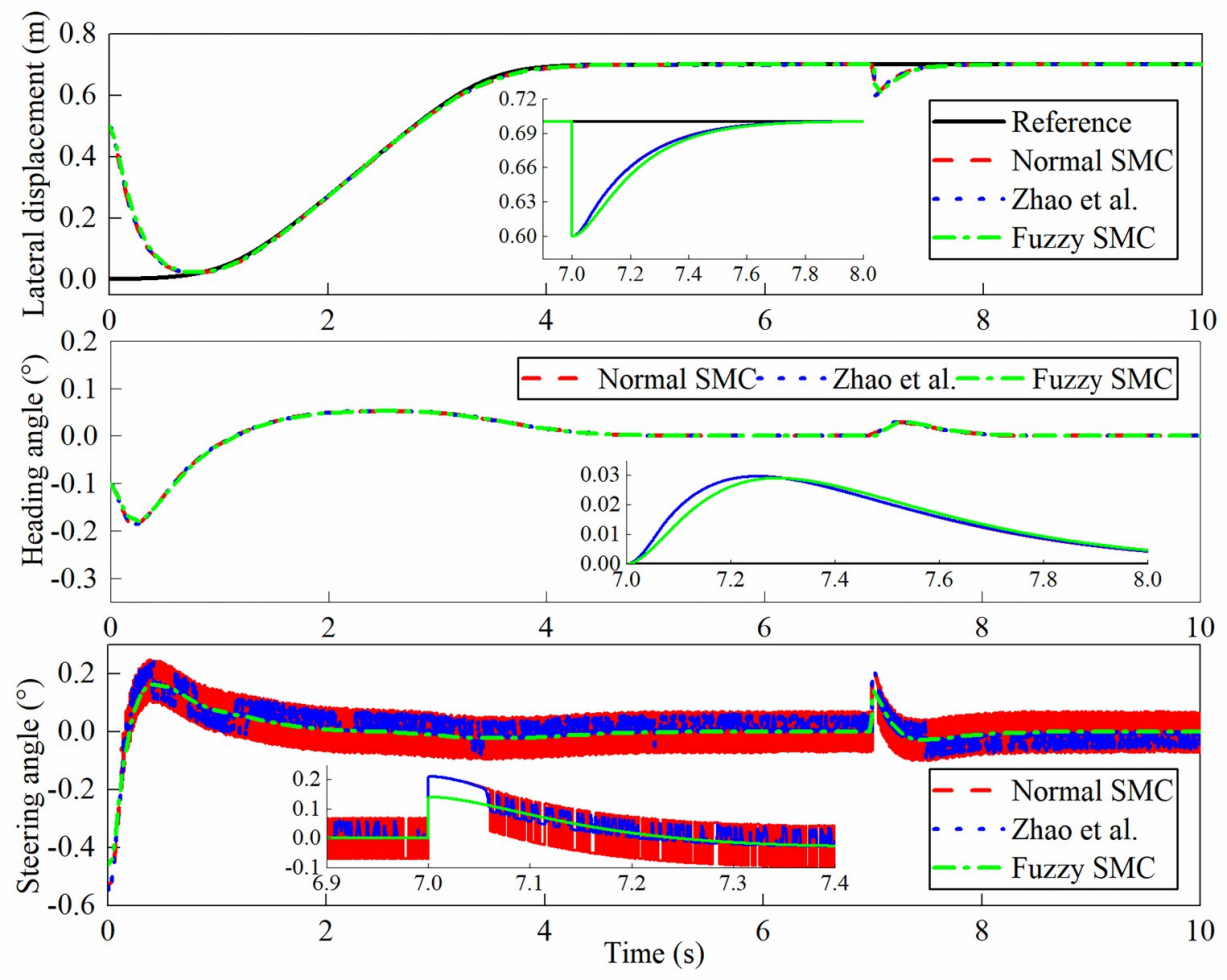
Lateral displacement, heading angle, steering angle with disturbance.

The simulation outputs are shown in Figs 13–16. In this case, Fig 13 shows the lateral displacement, heading angle and steering angle. Fig 14 illustrates the lateral error and heading angle error. Fig 15 demonstrates the three sliding mode surfaces. Fig 16 presents the lateral displacement, heading angle and steering angle with disturbance. In Figs 13 and 16, the initial lateral deviation between trajectory and tractor is 0.5m, and the initial heading angle is -0.1°. The three controllers are extremely fast and are not affected by the changing velocity. In comparison, the rapidity of the three controllers is almost the same, but it can be seen from the local magnification graph that the modified sliding mode controller proposed by Zhao *et al*. has better performance than the normal sliding mode controller, which can weaken the jitter of the system better but does not eliminate it completely, while the fuzzy sliding mode controller solves the system jitter problem completely. As can be seen in Figs 14 and 15, all sliding mode surfaces constructed in the control process are asymptotically stable.

To show the performance of disturbance rejection of the designed system, a negative step input of size 0.1 to the desired trajectory at t = 7s. Fig 16 clearly reflects the dynamic response of the tractor with disturbance. It can be seen that the displacement and steering of the system are less affected by the disturbance, and the system becomes stable after 1s, which indicates that the fuzzy sliding mode controller has a good disturbance rejection ability.

In order to quantify the performance of the three control methods, performance index such as the variance (VAR), root mean square (RMS) values and coefficient of variation (CV) are used to evaluate the three methods. The calculated results are shown in Table 2. The smaller the index value means the better the controller performance. Although the deviation of the index is small, it also indicates that the fuzzy sliding mode controller has better performance than the ordinary sliding mode controller.

**Table 2:** Performance measures for the three methods.

Method	VAR	RMS	CV
Normal			
Normal SMC	0.0081	0.0907	9.3249
Zhao *et al*.	0.0033	0.0600	3.0674
Fuzzy SMC	0.0027	0.0523	5.5846
Perturbed			
Normal SMC	0.0083	0.0916	9.0205
Zhao *et al*.	0.0036	0.0617	4.3184
Fuzzy SMC	0.0028	0.0535	5.8781

**Fig 17:**
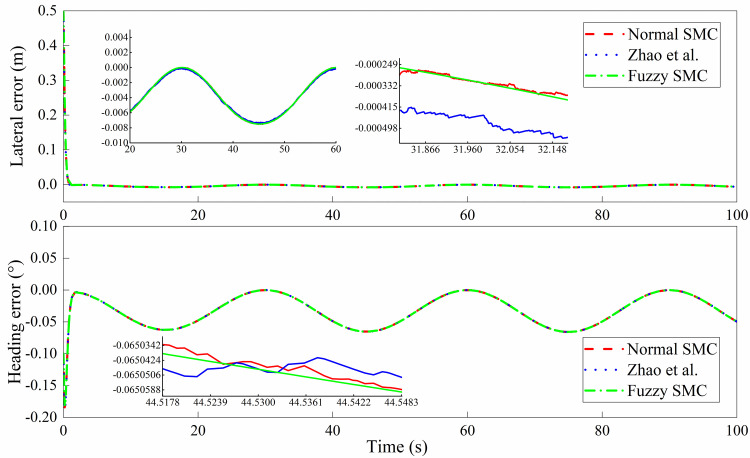
Lateral error, heading error with continuous channel change.

**Fig 18:**
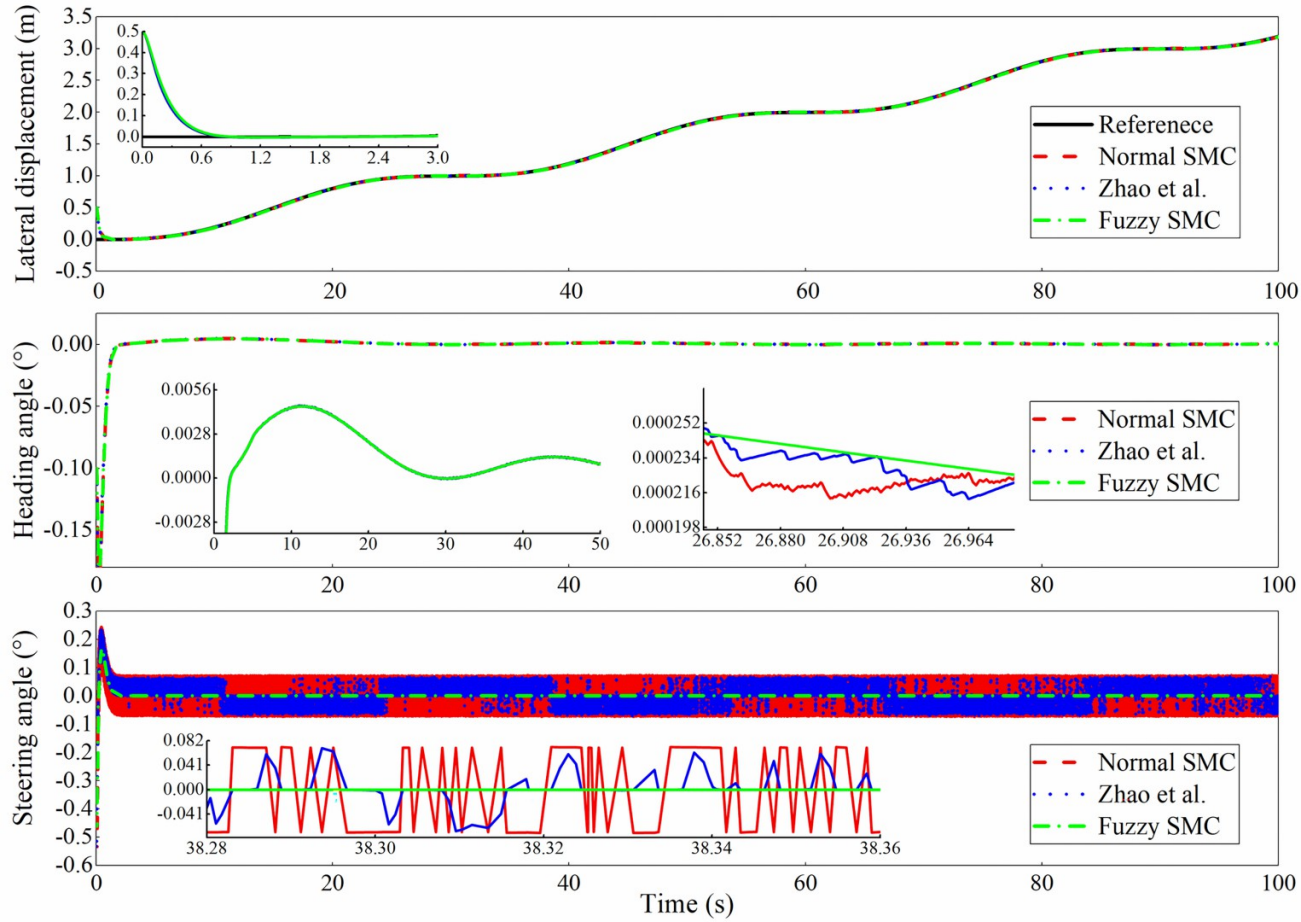
Lateral displacement, heading angle, steering angle with continuous channel change.

To further prove the effectiveness of the improved controller, a continuous lane change condition are performed. In this case, both the tractor system parameters and the controller parameters are kept the same as before. The results of the continuous channel change test are shown in Figs 17 and 18. Similarly, the deviation value between the initial position of the tractor and the reference trajectory is set to 0.5m. As can be seen in Fig 17, all three controllers enable the tractor to quickly track to the desired reference trajectory and maintain excellent tracking accuracy during continuous trajectory changes. The same conclusion can be drawn from the partial magnification diagram that the sliding mode controller improved based on fuzzy rules is jitter-free, especially for the steering angle. The trajectory tracking error and heading angle error are within the acceptable range.

It can be seen from the above analysis results that the designed fuzzy sliding mode control system can accurately and quickly track the desired trajectory. The error of the lateral deviation can converge to zero in a short time and the overshoot is small in the different cases of the tractor. With superior tracking performance and disturbance rejection, the designed control method is feasible and effective for agricultural tractor.

## Conclusion

A trajectory tracking control system for agricultural tractor aided by an electric power steering mechanism was presented in this paper. For the adopted steering system structure, the steering tracking controller hardware circuits were designed including torque detection circuit, relay control circuit, current control and sensing circuit. To realize accurate trajectory tracking, a control strategy based on the established three DOF model and steering system model of the tractor was proposed, in which fuzzy sliding mode controller was designed to control the lateral displacement of the tractor and a PID controller based on internal mode control and minimum sensitivity to control the front wheel angle. Finally, the performances of the designed control strategy were verified in comparison with the other two methods, and performance indexes VAR, RMS land CV were introduced to evaluate the three control methods. The simulation results showed that the proposed control system for an autonomous tractor has satisfactory trajectory tracking ability and favorable disturbance rejection ability.
